# ASME B89.4.23 Performance Evaluation Tests and Geometry Errors in X-Ray Computed Tomography Systems

**DOI:** 10.6028/jres.126.042

**Published:** 2022-01-20

**Authors:** Bala Muralikrishnan, Meghan Shilling, Vincent Lee

**Affiliations:** 1 Sensor Science Division, National Institute of Standards and Technology, Gaithersburg, MD 20899, USA

**Keywords:** ASME B89.4.23, cone-beam X-ray computed tomography, geometry errors, performance evaluation

## Abstract

A documentary standard produced by the American Society of Mechanical Engineers (ASME) for performance evaluation of industrial X-ray computed tomography (XCT) systems for dimensional measurements was released in early 2021. This standard, ASME B89.4.23-2020, specifies test procedures that may be performed to determine whether a system meets the manufacturer’s accuracy specifications for acceptance before or after purchase, or for periodic reverification. While there are some core testing requirements in the standard, there is also some flexibility, allowing for a variety of testing configurations that meet the requirements of the standard. It is important that the chosen testing configuration be sensitive to the different systematic sources of error in XCT systems to provide confidence that the system will meet the manufacturer’s accuracy specifications for measurements performed by the user subsequent to testing. In this paper, we provide guidance on how to optimally apply the ASME 89.4.23 standard in industry to achieve high sensitivity to geometry errors in cone-beam XCT systems. Through simulation studies, we present some examples of testing configurations that meet the requirements of the ASME B89.4.23 standard and discuss their sensitivity to geometry errors of the detector and the rotation stage. We show that there are some testing configurations that achieve maximal sensitivity to these errors, while other configurations do not capture these error sources with adequate sensitivity.

## 1 Introduction

X-ray computed tomography (XCT) is increasingly used for dimensional inspection of internal and delicate features that cannot be measured using traditional tactile probe–based Cartesian coordinate machines (CMMs) [1–5]. To unify accuracy specifications from the different manufacturers and allow easier comparison, the Association of German Engineers and the Association of German Electrical Engineers, the VDI/VDE, released a series of guidelines [6–8] for specifying and testing the accuracy of industrial XCT systems. In the United States, the American Society of Mechanical Engineers (ASME) Committee B89.4 on Coordinate Metrology established a working group in 2015 to develop a documentary standard for performance evaluation of these systems. That working group, of which the first and second authors were members, completed their task in late 2020, leading to the publication of the ASME B89.4.23 [9] standard in early 2021. This comprehensive performance evaluation standard prescribes test procedures that may be performed by the user or the manufacturer for acceptance of a new system, *i.e.*, to determine whether the new system meets accuracy specifications provided by the manufacturer. The test procedures may also be performed by the user on a periodic basis as a reverification process to ensure the system continues to perform as designed. In an independent effort, Technical Committee (TC) 213 within the International Organization for Standardization (ISO) is currently working on a standard for performance evaluation of XCT systems [10]. Note that there are other documentary XCT standards developed by ASTM International [11–12] and ISO [13], but these address image quality and are primarily intended for nondestructive evaluation, not dimensional measurements.

While the ASME B89.4.23 provides some core requirements for testing, there is also some flexibility to allow for a variety of testing configurations that meet the requirements of the standard. To provide confidence that the system will meet manufacturer’s accuracy specifications during regular use subsequent to the testing process, it is important to ensure that the testing configuration chosen by the user or the manufacturer is sensitive to all significant sources of error in XCT systems.

There are many error sources in XCT systems, see Refs. [7, 14] for more information. In this paper, we address the sensitivity of different testing configurations to geometry errors (such as detector and rotation stage errors) that may be present in a typical cone-beam XCT system. Other error sources that are commonly present in XCT systems, such as beam-hardening, scatter, cone-beam artifacts, source drift, non-monochromatic nature of X-ray wavelength, and detector nonlinearities, are outside the scope of this study. We present examples of several testing configurations and, through simulation studies, capture the sensitivity of each configuration to the different geometry errors. We make recommendations on optimal choices of testing configurations that provide maximum sensitivity to the geometry errors.

The rest of the paper is organized as follows. We provide a brief review of related literature in Sec. 2, present an overview of the different geometry errors in Sec. 3, discuss the requirements of the ASME B89.4.23 standard in Sec. 4, discuss all aspects pertaining to the simulation study in Sec. 5, and present conclusions in Sec. 6.

## 2 Literature Review

There is considerable literature on error characterization of XCT systems. The focus of this paper is on performance evaluation, *i.e.*, on the methods used to assess dimensional measurement accuracy of commercial systems. Therefore, we limit our literature review to that topic, particularly using calibrated reference objects, as that is the approach adopted by documentary standards committees.

Lettenbauer *et al*. [15] discussed methods to characterize the accuracy of XCT systems at a time when there were no published documentary standards for evaluating their performance. Among the different reference objects, they described the use of a calibrated test piece consisting of several ruby spheres mounted on carbon fiber shafts. This reference object has since been referred to as a sphere-forest and is being considered within ISO TC213 as an option for verifying the performance of XCT systems. Su *et al*. [16] investigated various designs and materials for the sphere-forest and suggested that the stems should be made of ceramic instead of carbon fiber so that they are more stable, enabling lower uncertainty in CMM measurements to establish reference values of the sphere center positions. Su *et al*. further noted that the distribution of spheres in the standard is not a significant concern, but that the selection of test lengths is important in the verification of XCT systems. Fujimoto *et al*. [17], Welkenhuyzen *et al*. [18], and Villarraga-Gómez *et al*. [19] also reported on the use of the sphere-forest for performance evaluation of an XCT system. Fujimoto *et al*. [17] noted that the near-planar nature of the reference object means that it is advantageous to make the XCT measurements with the reference object positioned at different heights.

Hiller *et al*. [20] described the use of a calibrated ball bar to estimate length measurement errors in an XCT system through eight measurements made in different orientations of the ball bar in the measurement volume. Léonard *et al*. [21] described the use of a novel reference object composed of four spheres arranged in a tetrahedron, where the spheres make contact with each other. In an intercomparison study reported by Carmignato [22], one of the reference objects that was measured by the different participating laboratories was a tetrahedral structure with carbon fiber reinforced plastic (CFRP) tubes forming the sides and the spheres representing the vertices. Müller *et al*. [23] and Moroni and Petrò [24] used a ball and plate design composed of ruby spheres mounted on a CFRP plate for evaluating the performance of XCT systems, where the inter-sphere distances were calibrated using a CMM. Villarraga-Gómez and Smith [25] used a hole plate, which is similar in concept to a ball plate, where the distances between holes in the midplane of the plate were calibrated using a CMM. Ferrucci *et al*. [26–27] and Muralikrishnan *et al*. [28] reported on reference objects consisting of spheres mounted on a hollow cylindrical framework to assess errors in XCT systems. A summary of different reference objects used for evaluating the performance of XCT systems was provided by Müller [29]. Because the work we describe in this paper is focused on geometry errors in XCT systems, we note that this topic has been explored by Kumar *et al*. [30] and Ferrucci *et al*. [26–27, 31]. Both sets of studies considered the effect of detector geometry errors on dimensional measurements by considering a simulated reference object composed of spheres. For a detailed review of geometry errors in XCT systems, see Ferrucci *et al*. [32] and Carmignato *et al*. [14].

Given the number of different methods proposed for performance evaluation, there is clearly a need to adopt standardized procedures that will provide users with confidence that the system meets manufacturer specifications. Lettenbauer *et al*. [15] discussed this lack of standardization in an early publication. The progress and updates in the area of standardization have since been reported by Takatsuji *et al*. [33], Bartscher *et al*. [34], and Shakarji *et al*. [35].

## 3 Geometry Error Sources in XCT Systems

**Fig. 1:**
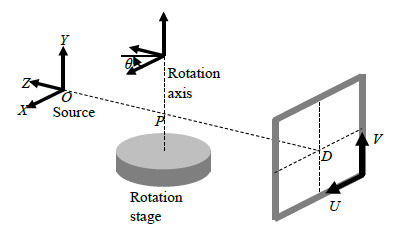
Schematic of setup and coordinate system definition.

Figure 1 shows a schematic of a cone-beam XCT system and the coordinate system employed in this paper. An ideal point source is located at *O*. The line joining the source orthogonally intersects the axis of rotation of the rotation stage at point *P*. This line intersects the detector at point *D*. The *Z* axis is coincident with this line, with the positive direction pointing away from the detector as shown in Fig. 1. The *Y* axis is parallel to the axis of rotation. In an ideally aligned instrument, the *Z* axis intersects the plane of the detector orthogonally at the detector’s geometrical center. By definition, the global *X* and *Y* coordinates of *D* are zero for an ideally aligned system. Point *P* is also the center of the measurement volume. The location of point *D* in the detector coordinate system (*U*-*V*) is given by (*u*, *v*) and is assumed to be known from prior calibration. In an ideal instrument, the *U* and *V* axes of the detector coordinate system are respectively parallel to the *X* and *Y* axes of the global coordinate system.

The geometry error sources discussed in this paper can be grouped into two categories—position/pose errors and rotation stage errors (see Table 1). Position/pose errors include the three location errors of the detector along each Cartesian axis of the global coordinate frame, three angular errors of the detector about the same axes, and the *Z* location error of the rotation stage; see Refs. [36–37] for more information. Note that the *Z* axis is defined as the line orthogonally intersecting the source and the axis of rotation; thus, if the rotation stage were located at an unexpected position along the *X* and *Y* axes, that would be reflected as a detector position/pose error, not as an error of the stage along those axes. Thus, there is no rotation stage location error along the *X* and *Y* axes. Therefore, by definition, the *X* and *Y* coordinates of the rotation stage location *P* are always zero.

**Table 1:** XCT instrument geometry error sources.

Position/Pose (Detector Geometry and Rotation Stage *Z* Location) Errors	Rotation Stage Errors
• Detector *X* location error • Detector *Y* location error • Detector *Z* location error • Detector *X* rotation error • Detector *Y* rotation error • Detector *Z* rotation error • Rotation stage *Z* location error	• Axial error along *Y* axis • Radial error along *X* axis • Radial error along *Z* axis • Wobble error about *X* • Wobble error about *Z* • Scale errors in the indexing angle
Each of the above terms is a scalar, so there are seven error sources in total.	There are eight components (four cosine and four sine orders) considered in this study for each of the six terms listed above (axial, radial *X*, radial *Z*, wobble *X*, wobble *Z*, and scale), for a total of 48 error sources.
Total of 55 error sources considered.	

The rotation stage errors describe the intrinsic errors of the stage such as axial, radial, wobble, and scale (angular indexing) errors. These are all assumed to have harmonic components and therefore are represented as sine and cosine functions of the nominal rotation stage indexing angle *θ*, *i.e.*, of the form $a\sin\left(n\theta \right)\;$and $a\cos\left(n\theta \right),\;$where *a* represents the amplitude or magnitude of the error, and *n* is the order of the harmonic. Table 1 presents a list of the error sources considered. In this study, we considered harmonics of orders one through four for the axis of rotation errors, since low orders are generally dominant in rotation stages. Thus, there are six detector error sources, one error source associated with the *Z* location error of the rotation stage, and 48 error sources associated with the rotation axis, for a total of 55 error sources. We previously considered the effect of the first 10 orders (*i.e.*, *n* = 1 to 10) on dimensional measurements for one position of the rotation stage and detector in Ref. [37]. We provided a more detailed discussion and plots covering many rotation stage and detector positions for the first four orders of rotation stage errors in Refs. [38–39].

Before we proceed, we note that among the geometry errors, position/pose error sources are generally the more significant error sources, while the contributions of rotation stage errors, especially those from high-quality precision stages, are expected to be substantially smaller. For this reason, we discuss the effect of these error sources separately in later sections.

## 4 ASME B89.4.23 Requirements

The ASME B89.4.23 standard prescribes three types of tests to be performed as part of the acceptance/reverification procedures. Using spheres as metrological geometric elements, the standard requires the determination of sphere center-to-center distance error, sphere form error, and sphere size error for spheres located at carefully chosen positions in the measurement volume. In this paper, we focus on sphere center-to-center distance error and sphere form error only. The standard describes testing requirements for the case of one, two, and three rated material classes, chosen from plastic, aluminum, or steel. In this paper, we focus on the case of one rated material only. We do not consider material penetration effects in this paper, so the choice of the material class (plastic, aluminum, or steel) is not relevant. The standard also considers the introduction of obstructive bodies to assess their influence on dimensional measurements. In this paper, we do not consider the effect of obstructive bodies because our focus is purely on dimensional measurement sensitivity to instrument geometry error sources.

### 4.1 Length Tests

The requirements of the ASME B89.4.23 standard for the length (sphere center-to-center distance) tests for the case of one rated material include:

(1) A total of 112 test lengths shall be measured equally distributed among four planes, *i.e.*, 28 lengths per plane.
(2) One of the four planes shall be oriented horizontally, one shall be oriented vertically, and one shall be oriented diagonally. The orientation of the fourth plane may be specified by the user.
(3) The 28 lengths in each plane shall be realized using a minimum of eight spheres. In the cases of the horizontal, vertical, and diagonal planes, six of the 28 lines shall be along specified orientations described in the standard (see Fig. 2). If there are more than eight spheres in a plane, the 28 lengths shall be identified prior to testing.
(4) The four planes shall be distributed among two magnifications (where magnification *M* is the ratio of source–detector and source–rotation stage distances), so either one, two, or three planes shall be measured at one magnification (magnification *M*_1_ in this text), and the remaining plane(s) shall be measured at the other magnification (magnification *M*_2_).
(5) At least one of the lines in each plane shall be at least 66% of the longest possible length in that plane, as defined by the size of the measurement volume.

The physical reference object used to realize the planes may be two-dimensional (2D) with just one plane or three-dimensional (3D) with two or three planes. Multiple scans may be necessary to realize the above requirements.

**Fig. 2:** Schematic of mandatory lines in each of the three mandatory planes in the ASME B89.4.23 standard: (a) horizontal plane, (b) vertical plane, (c) diagonal plane oriented approximately 45° to the axis of rotation. **Table:** 

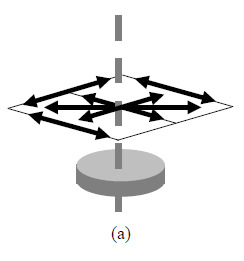	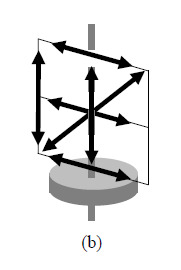	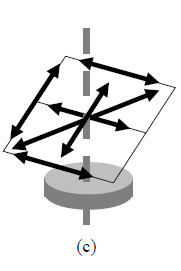
(a)	(b)	(c)

### 4.2 Form Tests

The form error, calculated from an unconstrained least-squares best-fit sphere, is calculated for each of the eight spheres in each of the four planes, for a total of 32 form error values. If there are more than eight spheres in a plane, then eight spheres are to be identified prior to testing.

## 5 Simulation Approach and Results

### 5.1 Single-Point Ray Tracing (SPRT)

The results described in this paper were generated using the single-point ray tracing (SPRT) method described in Refs. [36–37]. The method is based on applying multi-view geometry principles to approximate the forward- and back-projection steps, which correspond to the radiographic acquisition and reconstruction steps in XCT, respectively, thereby avoiding the computational burden of generating a full radiographic data set and of reconstructing a full gray-value voxel model, as well as the subsequent dimensional evaluation. The method only applies to spherical test objects, but that is not a constraint here because a sphere is the metrological geometric element considered in the ASME B89.4.23 standard.

The concept of the SPRT technique is as follows. We consider a single geometry error source, such as detector *X* location error, for a given SPRT simulation. In the presence of this error source, we forward project the 3D coordinates of each sphere center in the reference object onto the detector and determine the corresponding center projection coordinates in the detector’s image frame. The detector is assumed to be continuous, *i.e.*, not pixelated. As the stage makes a full rotation, each projected sphere center traces a locus on the detector. In the back-projection step, we assume ideal geometry, *i.e.*, no geometry errors in the system. This back-projection step mimics an actual reconstruction in which the system would be unaware of the presence of geometry errors. For each sphere, we consider the rays from the source to the detector for each of the rotation stage positions, and through a least-squares minimization, we determine the location of the center of the sphere in the measurement volume. Thus, the center of the sphere in the presence of the imposed geometry error is determined without any radiographs or reconstruction. This least-squares–based minimization process is sequentially performed for all spheres in the reference object. This method has a dedicated purpose of estimating the effects of geometry error sources on sphere-based objects. It has been validated and proven to be a faster and more practical alternative to simulating the full XCT radiographic acquisition and subsequent tomographic reconstruction.

To estimate form error, circles consisting of 120 equally spaced points are constructed normal to each ray connecting the source and the detector, with their centers located on the previously identified least-squares centers. This is performed for each angular position of the stage as it rotates, and therefore the circles at different rotational angles form a 3D point cloud. The diameters of each of these circles are equal to the diameters of the spheres in the reference object. The points lying in the interior of a convex hull generated from the resulting point cloud are truncated, and only the outer points are used for form error calculation. Form error is defined as the difference between the maximum and minimum deviations between an unconstrained least-squares best-fit sphere and the point-cloud data. While form error is sensitive to outliers, it is not a concern in this study because we do not consider the effect of random noise. The process described above is individually performed for all geometry errors associated with the detector and the rotation stage.

### 5.2 Sensitivity Definition

We use the term “sensitivity” extensively in this paper. In fact, there are two types of sensitivities we consider—distance error sensitivity and form error sensitivity. We define them here for clarity. Distance error sensitivity is the error in the distance between a pair of sphere centers for unit magnitude of a geometry error source. Form error sensitivity is the error in the form of a given sphere for unit magnitude of a geometry error source. These are expressed in units of mm/mm or mm/°, depending on the unit of the error source.

As an example, consider the case where the rotation stage and detector distances (*d* and *D*, respectively) from the source are 400 mm and 1177 mm, respectively. Let the side length (*s*) of a square detector be 190 mm. Then, from Ref. [36], 0.1 mm of simulated detector *Z* location error will result in a distance error of 0.006 mm for the long body diagonal, so the distance error sensitivity for that sphere pair is 0.06 mm/mm. Also, from Ref. [36] and for the same values of *d*, *D*, and *s*, 0.2° simulated error in detector rotation about the *Z* axis will result in a form error of 0.15 mm for a sphere located farthest from the axis of rotation and in the highest or lowest horizontal plane, so the form error sensitivity for a sphere in that location is 0.75 mm/°.

The calculation of a sensitivity coefficient assumes that the sphere center-to-center distance error and sphere form error are linearly related to the introduced geometry errors. For any given geometry error described in Table 1, we have performed (but not reported here) simulations for different magnitudes of the introduced geometry error to ensure that the center-to-center distance error and sphere form error do in fact have a linear relationship. See Refs. [36–38] for information on the magnitude of the imposed geometry errors used in the calculation of the sensitivities.

### 5.3 Testing Configurations Considered

**Fig. 3:**
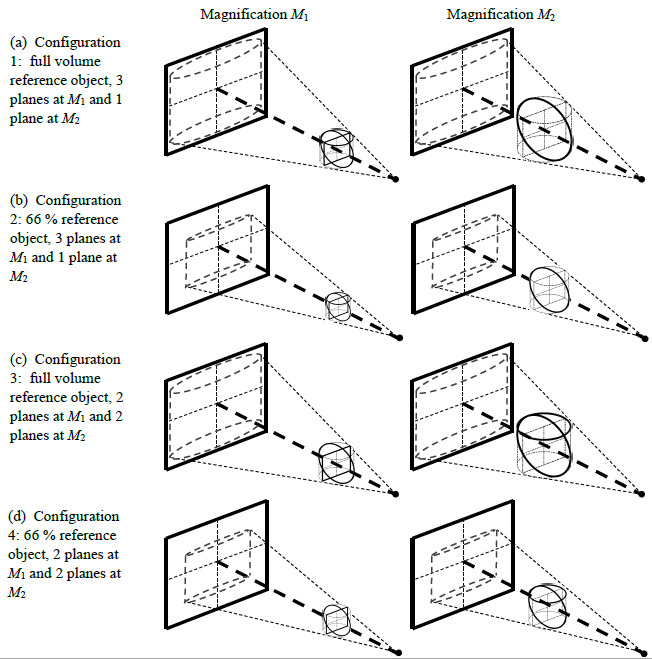
Schematic showing the four configurations described in Table 2: (a) full volume reference object (notice that it almost fills the detector) meeting the requirements of the ASME B89.4.23 standard by measuring three planes (horizontal, vertical, and diagonal) at *M*_1_ and one plane (diagonal shown in this example) at *M*_2_, (b) a reference object 66% in size (notice it only partially fills the detector) meeting the requirements as described in part (a), (c) full volume reference object meeting the requirements by measuring two planes (vertical and diagonal shown in this example) at *M*_1_ and two planes (horizontal and diagonal shown in this example) at *M*_2_, and (d) a reference object 66% in size meeting the requirements as described in part (c).

There are numerous testing configurations possible that meet the requirements listed in the ASME B89.4.23 standard. Table 2 lists the four configurations explored in this simulation study. Configurations 1 and 2 were selected to capture the two extreme conditions for object size (*i.e.*, the largest possible, limited by detector size [which we assume to be square], and the smallest allowable, limited by the 66% length requirement) that meet the requirements of the standard for the case of a three-plane reference object at magnification *M*_1_ and a single-plane reference object at magnification *M*_2_. Configurations 3 and 4 were selected to capture the two extreme conditions that meet the requirements of the standard for the case of a two-plane reference object at magnification *M*_1_ and a two-plane reference object (or two single-plane reference objects) at magnification *M*_2_. Figure 3 provides a visual schematic of the four testing configurations. Note that the ASME B89.4.23 standard requires the measurement of a line that is either coincident with the axis of rotation (for a vertical plane) or intersecting the axis (for horizontal and diagonal planes), so, in our simulations, the reference object is always centered in the measurement volume.

**Table 2:** Testing configurations.

Configuration	Magnification *M*_1_^a^	Magnification *M*_2_
1	Reference object scaled to fill the full volume^b^ to perform the ASME B89.4.23 tests using a three-plane measurement strategy at this magnification.	Reference object scaled to fill the full volume using a single-plane measurement strategy at this magnification.
2	Reference object^c^ scaled to 66% of the full volume using a three-plane measurement strategy at this magnification.	Reference object scaled to 66% of the full volume using a single-plane measurement strategy at this magnification.
3	Reference object scaled to fill the full volume using a two-plane measurement strategy at this magnification.	Reference object scaled to fill the full volume using a two-plane measurement strategy at this magnification.
4	Reference object scaled to 66% of the full volume using a two-plane measurement strategy at this magnification.	Reference object scaled to 66% of the full volume using a two-plane measurement strategy at this magnification.

^a^ *M* is the magnification, defined as the ratio of source-detector and source–rotation stage distances, where subscript 1 and 2 indicate the two testing positions in the measurement volume.

^b^ Full volume reference object refers to an object that is scaled to fill 98% of the detector; this includes the diameter of the spheres, which was chosen to be 10% of the diameter of the cylinder formed by the sphere centers.

^c^ The reference object is always centered with respect to the measurement volume to meet the requirements of the ASME B89.4.23 standard.

There are two modes of XCT system operation—fixed detector and moving detector modes. In the case of fixed detector systems, the requirement of performing tests at two magnifications can be realized by moving the rotation stage. In the case of moving detector systems, this requirement can be met by moving either or both the detector and the rotation stage. Thus, a measurement volume of a certain size can be realized through many combinations of detector and rotation stage positions, and this is also noted in ASME B89.4.23.

In the case of fixed detector systems, it is important to test for all detector and rotation stage geometry errors at one of the magnifications. As the rotation stage is moved to achieve a different magnification, it is assumed that the rotation stage errors (axial, radial, wobble, and scale) do not change. Because the rotation stage has moved along the *Z* direction, it is necessary to test for the *Z* location error of the stage at this magnification. While the detector position and orientation do not physically change, it is possible that the rotation stage has translated along the *X* and *Y* axes, and because of the way the coordinate system is defined, this stage translation appears as a change in the detector position and pose (as we described in Sec. 3). Thus, it is necessary to test for detector position/pose errors at this magnification.

In the case of moving detector systems, again, it is important to test for all detector and rotation stage geometry errors at one of the magnifications. As the rotation stage and/or the detector is moved, it is important to test for detector position/pose errors, and the rotation stage *Z* location error at the second magnification. As before, it is assumed that the rotation stage errors (axial, radial, wobble, and scale) do not change as the rotation stage is moved. Because the testing requirements are essentially the same for both the fixed detector and the moving detector modes, we only considered the moving detector mode in this study.

**Fig. 4:**
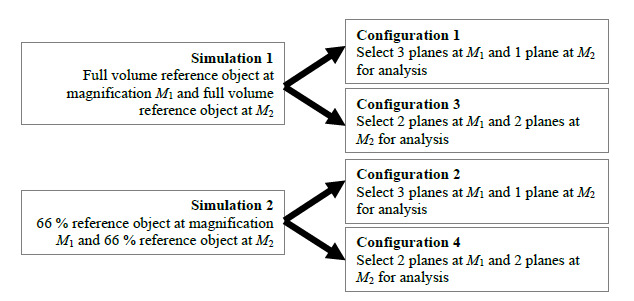
Schematic showing the two simulations and the four testing configurations.

Overall, we performed two simulations as part of this study, from which we extracted data that allow us to report on each of the four testing configurations in Table 2; see Sec. 5.4 for details on how two simulations provide data for all four testing configurations. Figure 4 shows a schematic of the two simulations providing information to assess the four testing configurations. The two magnifications considered are shown in Table 3. The first magnification, *M*_1_ = 3, was achieved through rotation stage and detector distances of 200 mm and 600 mm, respectively, from the source. The second magnification, *M*_2_ = 1.5, was achieved through rotation stage and detector distances of 800 mm and 1200 mm, respectively, from the source. The overall conclusions of this paper would not change given other choices for rotations stage and detector distances (and therefore other magnifications). The goal of this simulation exercise was to assess whether the objectives listed in Table 3 (and discussed earlier in this section) are being met and to make recommendations (see Sec. 5.7) on the optimal configuration for testing purposes.

**Table 3:** Simulation conditions and testing objectives.

	Magnification *M*_1_ = 3	Magnification *M*_2_ = 1.5
Conditions	*d* = 200 mm, *D* = 600 mm, *s* = 250 mm, see notes^a,b^	*d* = 800 mm, *D* = 1200 mm, *s* = 250 mm
Testing objectives	Sensitivity to all seven position/pose error sources Sensitivity to all 48 rotation stage errors	Sensitivity to all seven position/pose error sources

^a^ *d* is the rotation stage distance, *D* is the source-detector distance, and *s* is the side length of the square detector.

^b^ For the full volume reference object (fills 98% of the detector), the longest center-to-center length (*i.e.*, body diagonal) is 88.56 mm at magnification *M*_1_ and 192.16 mm at magnification *M*_2_.

### 5.4 Reference Object

**Fig. 5:**
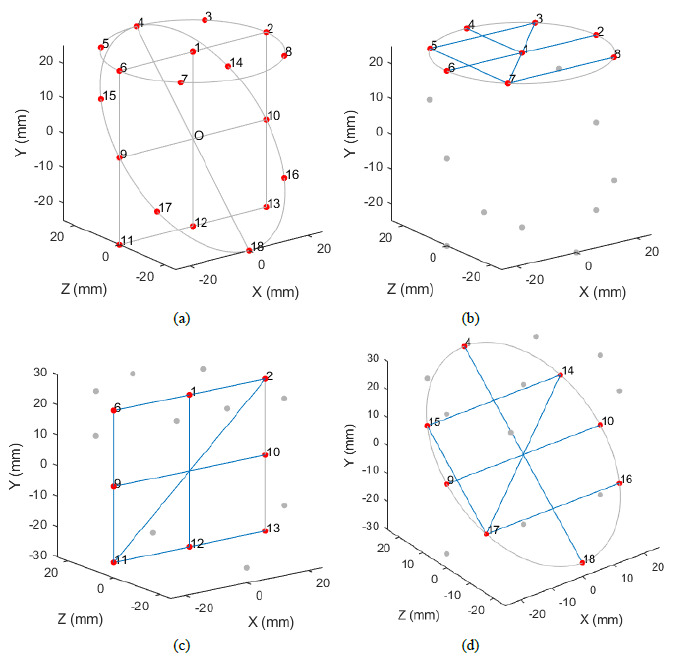
(a) Simulated reference object with 18 spheres distributed among three planes, (b) six mandatory lines in the horizontal plane, (c) six mandatory lines in the vertical plane, and (d) six mandatory lines in the diagonal plane.

The simulations were performed using a cylindrical reference object containing 18 spheres arranged as shown in Fig. 5(a) (only the sphere centers are shown, not the cylinder). Point *O* is the geometric center of the cylinder containing the spheres, *i.e.*, the point on the axis at half height of the cylinder. Spheres 1 through 8 lie on a horizontal plane, spheres 1, 2, 6, and 9 through 13 lie on a vertical plane, and spheres 4, 9, 10, and 14 through 18 lie on a diagonal plane (inclined at approximately 45°). Spheres 1 and 12 lie on a line coincident with the axis of the cylinder. Spheres 4 and 18 lie on a line coincident with one body diagonal of the cylinder. Figure 5(b–d) shows the six mandatory lines in each of the three planes that meet the requirements of the ASME B89.4.23 standard. The cylinder shown in Fig. 5 has a diameter and height of 50 mm. The cylinder is placed so that its axis is coincident with the axis of rotation, point *O* is coincident with the center of the measurement volume, and the vertical plane comprising spheres 2, 6, 11, and 13 is parallel to the plane of the detector at the first angular position of the rotation stage.

**Fig. 6:**
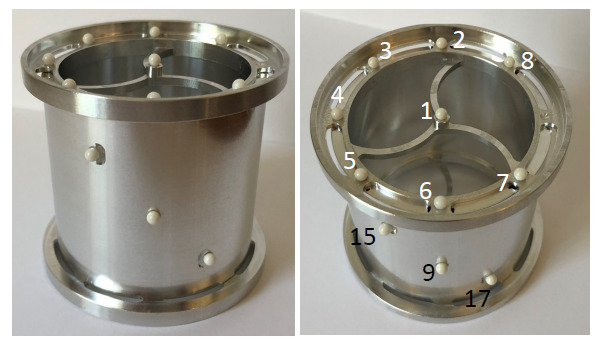
Physical embodiment of the reference object in Fig. 5 with 18 spheres distributed among three planes. Spheres 1–8 are arranged on a circle with a nominal diameter of 50 mm, while the spheres in the top and bottom planes are nominally 50 mm apart.

The arrangement of spheres shown in Fig. 5 is practically realizable as shown in Fig. 6 and may be calibrated using a CMM. In Fig. 6, the spheres are made of aluminum oxide ceramic material, while the support structure is made of aluminum. Spheres 1–8 are arranged on a circle with a nominal diameter of 50 mm, while the spheres in the top and bottom planes are nominally 50 mm apart. This reference object is sufficient to meet the needs at one magnification. We are currently in the process of manufacturing a second reference object that can be used at a different magnification. We plan on performing experimental measurements on an XCT system using this type of reference object in the near future.

SPRT simulations were performed on this 18 sphere reference object at two magnifications as shown in Table 3 to determine distance error and form error sensitivity for the 55 instrument geometry errors in Table 1. At each magnification, the diameter and height of the simulated cylinder were scaled based on the configuration as shown in Table 2, *i.e.*, either scaled to occupy the full measurement volume or scaled to occupy 66% of the measurement volume. In each case, point *O* was always coincident with the center of the measurement volume, the axis of the cylinder was coincident with the axis of rotation, and the vertical plane comprising spheres 2, 6, 11, and 13 was parallel to the plane of the detector at the first angular orientation of the rotation stage. The diameter of the spheres was set to 10% of the diameter of the cylinder.

Each of the two simulations (one for a full volume object and another for the 66% object) produced 55 distance error and 55 form error sensitivity values for:

• the horizontal plane in magnification *M*_1_,
• the vertical plane in magnification *M*_1_,
• the diagonal plane in magnification *M*_1_,
• the horizontal plane in magnification *M*_2_
• the vertical plane in magnification *M*_2_, and
• the diagonal plane in magnification *M*_2_.

Thus, for each of the two simulations, we produced six sets of 55 distance error and six sets of 55 form error sensitivity values. Note that for each of the 55 geometry error parameters, eight spheres in a plane resulted in 28 distance errors and eight form errors. We considered the maximum distance error and the maximum form error in the calculation of the sensitivity. Thus, the result of one simulation was a set of 55 distance error and 55 form error sensitivity values for each of the six planes. When studying configuration 1, we considered all three planes for magnification *M*_1_ but selected one plane for magnification *M*_2_. When studying configuration 3, we considered two planes in each of the two magnifications. Thus, this three-plane reference object provided sufficient information to model a three-plane, two-plane, and single-plane reference object that was selected.

### 5.5 Reference Sensitivity

The outcomes of the simulations were the distance and form error sensitivity values, as mentioned in Sec. 5.4. We refer to these as “test sensitivities,” since these values were obtained for a chosen testing configuration. Clearly, it is desirable to have larger sensitivity values from a testing perspective. However, the sensitivity values, by themselves, do not provide a significant amount of information. Our goal was to determine whether the chosen testing configuration could provide the largest possible sensitivity for each of the 55 geometry errors. Thus, in addition to the sensitivity values, we also required reference values against which we could compare the sensitivity values for the chosen testing configuration.

Reference sensitivities are the maximum achievable distance and form error sensitivity values for a given combination of distances *d* and *D* and detector size *s*. We described the calculation of reference sensitivity values for several combinations of *d*, *D*, and *s* in Refs. [38–39]. We briefly summarize the procedure here for completeness. We considered a reference object comprising 125 spheres distributed in the measurement volume. This large number of spheres provided adequate coverage of the measurement volume to identify the maximum possible sensitivity to a given error source. For chosen values of *d*, *D*, and *s*, we scaled the reference object so that it filled the detector. We considered one error source at a time. Using the SPRT method, we computed the effect of unit magnitude (for example, detector *X* location error of magnitude 1 mm or detector *Y* rotation error of magnitude 1°) of that error source on the distances between all pairs of spheres and on the form of all 125 spheres. We identified the pair of spheres that produced the largest distance error and the sphere that produced the largest form error. Because the introduced error was of unit magnitude, the largest distance error and the largest form error were the maximum sensitivity values for that error source. We repeated this process for all 55 error sources, thus determining the maximum distance and form error sensitivity values for each error source. Therefore, for the chosen values of *d*, *D*, and *s*, there were 55 values for reference distance error sensitivity and 55 values for reference form error sensitivity. In the next section, we describe how we used the test and reference sensitivity values to decide on the suitability of a chosen configuration for testing purposes.

Before we proceed, we present the following information that serves as a baseline for the results discussed in Sec. 5.7. Out of the seven position/pose error sources, five had a reference distance error sensitivity larger than a chosen threshold of 0.01 mm/mm or 0.01 mm/° at magnification *M*_1_, while four had a reference distance error sensitivity larger than the threshold at magnification *M*_2_. Also, out of the seven position/pose errors, five had a reference form error sensitivity larger than the threshold at magnification *M*_1_, while four had a reference form error sensitivity larger than the threshold at magnification *M*_2_. These values represent the maximum number of geometry errors we can possibly detect. When considering both distance error and form error, all seven can be detected with a sensitivity larger than the threshold at both magnifications. This information is summarized in Table 4. The table also shows similar information for rotation stage errors, where 32 and 26 rotation stage errors had reference distance error sensitivity larger than the threshold at magnifications *M*_1_ and *M*_2_, respectively, and all 48 had form error sensitivity larger than the threshold at both magnifications. The thresholds of 0.01 mm/mm and 0.01 mm/° were chosen so that realistic values of detector geometry error parameters would not lead to insignificantly small values for length errors and form errors.

In the case of position/pose errors, the table shows that both distance errors and form errors were sensitive to the same number of error sources. In the case of rotation stage errors, the table shows that form errors were sensitive to a larger number of error sources. Note that form errors are sensitive to outliers in the data, whereas distance errors, which are derived from a least-squares best fit to many points on the spheres, are less sensitive to outliers. Distance error measurements are also important in providing a link to the SI unit of length, the meter. Thus, distance error measurements are a critically important component of the testing process.

**Table 4:** Number of geometry error sources that can be detected (*i.e.*, sensitivity greater than threshold of 0.01 mm/mm or 0.01 mm/°) through distance error and form error measurements.

Position/Pose Errors^a^			
	Distance Error	Form Error	Distance and Form Error
Magnification *M*_1_	5	5	7
Magnification *M*_2_	4	4	7
Rotation Stage Errors^a^			
	Distance Error	Form Error	Distance and Form Error
Magnification *M*_1_	32	48	48
Magnification *M*_2_	26	48	48

^a^ Entries in table are based on a 125 sphere reference object.

### 5.6 Evaluation Metric

We first explain the evaluation metric using the horizontal plane for configuration 1 in Table 2. As mentioned in the previous section, the result of a simulation is one set of 55 test distance error sensitivity values and one set of 55 test form error sensitivity values based on the eight spheres in that plane. In addition to these, for the same combination of *d*, *D*, and *s*, we have a set of 55 reference distance error and 55 reference form error sensitivity values using a 125 sphere reference object, as described in the previous section. The metric employed to assess whether a chosen testing configuration is suitable for testing purposes is the sensitivity ratio. This is the ratio of the test sensitivity to the reference sensitivity values. Thus, for a given set of *d*, *D*, and *s*, there are 55 distance error sensitivity ratios and 55 form error sensitivity ratios. Note that we only calculated the ratio if the reference sensitivity values were larger than a threshold of 0.01 mm/mm or 0.01 mm/°.

Our objective was to identify the number of error sources that produced a distance or form error sensitivity ratio greater than or equal to 0.9. In an ideal situation, this sensitivity threshold condition will be met for seven position/pose errors and 48 rotation stage errors, indicating that the horizontal plane in configuration 1 is sensitive to all geometry errors for either distance error measurements or form error measurements, or both. The tables in Sec. 5.7 show the number of geometry errors that can be detected at a sensitivity ratio of 0.9 or higher.

In addition to analyzing the sensitivities from a single plane, we were also interested in analyzing the sensitivities from a collection of planes, since there may be more than one plane at a given magnification (to meet the criteria listed in Sec. 4.1). In that case, for each of the seven position/pose errors and the 48 rotation stage errors, we calculated the distance and form error sensitivity for each plane and considered the larger value as the test distance and form error sensitivity. We then calculated the sensitivity ratios as described earlier.

### 5.7 Results

#### 5.7.1 Configuration 1

In this configuration, we considered three planes at magnification *M*_1_ and one plane at magnification *M*_2_, scaled so that they occupied the full measurement volume. Table 5 shows that the eight spheres in the horizontal plane provided distance error sensitivity to two out of the seven position/pose errors, while they provided form error sensitivity to five out the seven errors. If we consider sensitivity to either distance or form error, the eight spheres in the horizontal plane provided sensitivity to six out of the seven position/pose errors. Note that this value, six, is not simply the sum of the preceding two entries in that row, two and five, because some error sources are sensitive to both distance and form errors. The eight spheres in the vertical and diagonal plane produce sensitivity to seven and six errors, respectively. If we consider all distance errors from the three planes, they are sensitive to five position/pose error sources. If we consider the form error of spheres in all three planes, they are sensitive to five error sources. If we consider all distance and form errors from all three planes, then this configuration achieves the desired objective of providing sensitivity to all seven position/pose geometry errors. Following the same argument, if we consider all distance and form errors from all three planes, this configuration achieves the desired objective of providing sensitivity to all 48 rotation stage geometry errors.

As noted in Table 3, we were interested in testing for sensitivity to all seven position/pose errors at the second magnification. A horizontal plane does not provide sensitivity to rotation stage and detector *Z* location errors because of the absence of a long body diagonal in that plane (see Ref. [36] for sensitive test positions for this error source), while a diagonal plane does not provide sensitivity to detector rotation error about the *X* axis because it does not have a face diagonal at the top/bottom plane (again, see Ref. [36]). A vertical plane is desirable at this magnification because it provides sensitivity to all seven position/pose errors.

**Table 5:** Number of geometry error sources that can be detected at magnification *M*_1_ for configuration 1.

Position/Pose Errors (7 Total)			
	Distance Error	Form Error	Distance and Form Error
Horizontal plane	2	5	6
Vertical plane	4	5	7
Diagonal plane	3	5	6
All planes	5	5	7
Rotation Stage Errors (48 Total)			
	Distance Error	Form Error	Distance and Form Error
Horizontal plane	17	48	48
Vertical plane	10	45	46
Diagonal plane	7	45	45
All planes	24	48	48

#### 5.7.2 Configuration 2

In this configuration, we considered three planes at magnification *M*_1_ and one plane at magnification *M*_2_, scaled so that they occupied 66% of the measurement volume. Note that the reference object was centered in the measurement volume. Table 6 shows that none of the planes provided distance or form error sensitivity to any of the seven position/pose error sources. The table also shows that none of the planes provided distance error sensitivity to any of the 48 rotation stage error sources, while they did provide form error sensitivity to only a small number of those errors. Overall, when considering all three planes and both distance and form errors, only 6 out of the 48 rotation stage errors were captured with adequate sensitivity. In the second magnification, all three planes provided sensitivity to detector *X* location error. This error source produces a form error on a sphere in any location in the measurement volume, so all three planes are sensitive to this error source. However, none of the three planes provided sensitivity to the remaining detector position/pose errors and rotation stage *Z* location error. Thus, this testing configuration is not an optimal choice for the user.

We do recognize that maximum permissible error (MPE) specifications are typically expressed in the form *A*+*BL* (where *A* and *B* are constants, and *L* is the length), acknowledging the possibility that error sources are expected to scale with length. Thus, reducing the size of the reference object will likely result in corresponding reduction in the errors, and therefore in the sensitivity to those errors. Thus, as we reduce the size of the reference object to 66%, we also consider the case where the threshold for the sensitivity ratio is 0.9 × 0.66 = 0.594, *i.e.*, 59.4% of the maximum sensitivity. Table 7 shows the number of geometry errors that can be detected at magnification *M*_1_ for configuration 2 for this case. Only three out of the seven position/pose error sources and 43 out of the 48 rotation stage errors can be detected at a 59.4% threshold, suggesting that the length/form errors due to the geometry errors do not necessarily scale proportionately with the size of the reference object. In fact, in order to detect all 55 error sources at magnification *M*_1_, the threshold for the sensitivity ratio must be reduced to 0.4, which represents a substantial reduction in sensitivity as we scale the reference object to 66% of the measurement volume.

**Table 6:** Number of geometry error sources that can be detected at magnification *M*_1_ for configuration 2.

Position/Pose Errors (7 Total)			
	Distance Error	Form Error	Distance and Form Error
Horizontal plane	0	0	0
Vertical plane	0	0	0
Diagonal plane	0	0	0
All planes	0	0	0
Rotation Stage Errors (48 Total)			
	Distance Error	Form Error	Distance and Form Error
Horizontal plane	0	6	6
Vertical plane	0	6	6
Diagonal plane	0	6	6
All planes	0	6	6

**Table 7:** Number of geometry error sources that can be detected at magnification *M*_1_ for configuration 2 when the threshold for sensitivity ratio is 0.594.

Position/Pose Errors (7 Total)			
	Distance Error	Form Error	Distance and Form Error
Horizontal plane	0	1	1
Vertical plane	2	1	3
Diagonal plane	2	1	3
All planes	2	1	3
Rotation Stage Errors (48 Total)			
	Distance Error	Form Error	Distance and Form Error
Horizontal plane	6	43	43
Vertical plane	6	39	39
Diagonal plane	6	40	40
All planes	12	43	43

**Fig. 7:**
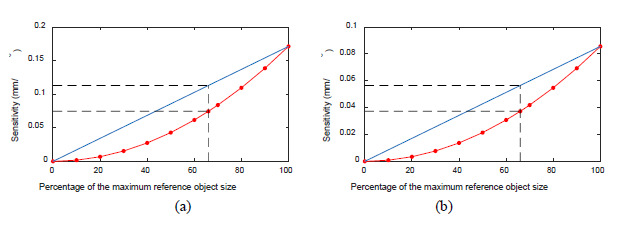
Distance error sensitivity as a function of reference object size (red line with markers) for (a) detector *X* rotation error and (b) first-order scale error in the angle of the rotation stage encoder. The blue lines show the anticipated sensitivity had the errors scaled proportionately with reference object size.

We examine this exaggerated drop in sensitivity with decreasing reference object size through the following examples. Consider the case of detector rotation error about the *X* axis. The line that produces the largest length error for this error source is a face diagonal in the horizontal plane, for example, between spheres 2 and 6 in Fig. 5(b), when the reference object is scaled to fill the detector. For the case of *d* = 200 mm, *D* = 600 mm, *s* = 250 mm, and for a 0.2° detector rotation error about the *X* axis, the error in that face diagonal is 0.0342 mm, *i.e.*, a sensitivity of 0.171 mm/°. This is the maximum sensitivity achievable for this error source at this location. We now scale the reference object down from 100% to 0% of the original size in steps of 10% and compute the errors in that length for the same 0.2° detector rotation error about the *X* axis. We also compute the errors for the case where the reference object is 66% of the original size. In each case, the reference object remains centered in the measurement volume, so the face diagonal is displaced towards the center as the object is shrunk. Figure 7(a) shows that the sensitivities are not linearly related to the size of the reference object. The sensitivity for an object that fills the detector (98% as mentioned in Sec. 5.3) is about 0.171 mm/°, the sensitivity for an object that is 66% in size is only 0.075 mm/°, *i.e.*, 44% of the maximum sensitivity. This represents a sensitivity ratio of 0.075/0.171 = 0.439, which is lower than our chosen threshold of 0.594. The anticipated sensitivity would have been 0.171 × 0.66 = 0.113 mm/°, *i.e.*, 66% of the maximum, had the errors scaled proportionately with reference object size (the blue line shows this anticipated behavior).

Figure 7(b) shows another example of this behavior. Here, we plot the distance error sensitivities as a function of reference object size in the presence of 0.05° first-order scale error in the angle encoder of the rotation stage. The line that produces the largest length error for this error source is a vertical line, for example, between spheres 6 and 11 in Fig. 5(c), when the reference object is scaled to fill the detector. For the case of *d* = 200 mm, *D* = 600 mm, *s* = 250 mm, and for a 0.05° scale error, the error in that vertical line is 0.0043 mm, *i.e.*, a sensitivity of 0.0856 mm/°. The sensitivity for an object that is 66% in size is only 0.0373 mm/°, which is 44% of the maximum sensitivity. This represents a sensitivity ratio of 0.436, which is lower than our chosen threshold of 0.594. The anticipated sensitivity would have been 0.0856 × 0.66 = 0.0565 mm/°, *i.e.*, 66% of the maximum, had the errors scaled proportionately with reference object size (the blue line shows this anticipated behavior). This sensitivity is nonlinear, and, as a result, the smaller reference object (scaled to 66%, centered on the measurement volume) is unable to detect the presence of this error with adequate sensitivity.

#### 5.7.3 Configuration 3

In this configuration, we considered two planes at magnification *M*_1_ and two planes at magnification *M*_2_, scaled so that they occupied the full measurement volume. When selecting pairs of planes from a set of three, there are three possible combinations. We present sensitivity information for each combination in Tables 8–10. Table 8 shows the number of geometry errors that can be detected at magnification *M*_1_ using a horizontal plane and a vertical plane. Table 9 shows the number of geometry errors that can be detected at magnification *M*_1_ using a horizontal plane and a diagonal plane. Table 10 shows the number of geometry errors that can be detected at magnification *M*_1_ using a vertical plane and a diagonal plane. Clearly, Tables 8 and 9 show that all seven position/pose errors and all 48 rotation stage errors can be captured when considering all three planes and both distance and form error sensitivity. However, Table 10 shows that the use of vertical and diagonal planes at magnification *M*_1_ is not desirable because it captures 47 out of the 48 rotation stage errors.

If we select the combination of a horizontal and a vertical plane (as shown in Table 8) at magnification *M*_1_, then there are two combinations for magnification *M*_2_ that are sensitive to all seven position/pose errors. These are a horizontal and a diagonal plane or a vertical and a diagonal plane. Two diagonal planes (one diagonal plane rotated about the axis of rotation with respect to the other) is not a good option because it does not include a face diagonal and is therefore not sensitive to detector rotation error about the *X* axis. If we select the combination of a horizontal and a diagonal plane (as shown in Table 9) at magnification *M*_1_, all possible choices at magnification *M*_2_ are suitable for testing, *i.e.*, a horizontal and a vertical plane, a vertical and a diagonal plane, and two vertical planes (one vertical plane rotated about the axis of rotation with respect to the other).

**Table 8:** Number of geometry error sources that can be detected at magnification *M*_1_ for configuration 3 for the case of a horizontal plane and a vertical plane.

Position/Pose Errors (7 Total)			
	Distance Error	Form Error	Distance and Form Error
Horizontal plane	2	5	6
Vertical plane	4	5	7
All planes	4	5	7
Rotation Stage Errors (48 Total)			
	Distance Error	Form Error	Distance and Form Error
Horizontal plane	17	48	48
Vertical plane	10	45	46
All planes	21	48	48

**Table 9:** Number of geometry error sources that can be detected at magnification *M*_1_ for configuration 3 for the case of a horizontal plane and a diagonal plane.

Position/Pose Errors (7 Total)			
	Distance Error	Form Error	Distance and Form Error
Horizontal plane	2	5	6
Diagonal plane	3	5	6
All planes	5	5	7
Rotation Stage Errors (48 Total)			
	Distance Error	Form Error	Distance and Form Error
Horizontal plane	17	48	48
Diagonal plane	7	45	45
All planes	20	48	48

**Table 10:** Number of geometry error sources that can be detected at magnification *M*_1_ for configuration 3 for the case of a vertical plane and a diagonal plane.

Position/Pose Errors (7 Total)			
	Distance Error	Form Error	Distance and Form Error
Vertical plane	4	5	7
Diagonal plane	3	5	6
All planes	5	5	7
Rotation Stage Errors (48 Total)			
	Distance Error	Form Error	Distance and Form Error
Vertical plane	10	45	46
Diagonal plane	7	45	45
All planes	13	47	47

#### 5.7.4 Configuration 4

In this configuration, we considered two planes at magnification *M*_1_ and two planes at magnification *M*_2_, scaled so that they occupied 66% of the measurement volume. Table 11 shows the number of geometry error sources that can be detected at magnification *M*_1_ using a horizontal plane and a vertical plane. The table also applies for the case of a horizontal plane and a diagonal plane, or for the case of a vertical plane and a diagonal plane. Clearly, they are all poor choices for a testing configuration, with none of the position/pose errors being detected and only 6 out of the 48 rotation stage errors being detected. Only the detector *X* location error is detected at magnification *M*_2_.

**Table 11:** Number of geometry error sources that can be detected at magnification *M*_1_ for configuration 4 for the case of a horizontal plane and a vertical plane.^a^

Position/Pose Errors (7 Total)			
	Distance Error	Form Error	Distance and Form Error
Horizontal plane	0	0	0
Vertical plane	0	0	0
All planes	0	0	0
Rotation Stage Errors (48 Total)			
	Distance Error	Form Error	Distance and Form Error
Horizontal plane	0	6	6
Vertical plane	0	6	6
All planes	0	6	6

^a^ The table also applies for the case of a horizontal plane and a diagonal plane or a vertical plane and a diagonal plane.

As in the case of configuration 2, we reduced the threshold for sensitivity ratio to 0.594 to assess whether the length/form errors due to the geometry errors scaled proportionately with the size of the reference object. Tables 12–14 show the number of error sources that can be detected at this sensitivity ratio threshold for the different choices for the two planes at magnification *M*_1_. Clearly, none of the choices shown in the tables can detect all seven position/pose error sources and all 48 rotation stage errors. If we reduced our threshold for sensitivity ratio to 0.4, we could detect all 55 error sources at magnification *M*_1_. However, this represents a substantial reduction in sensitivity as we scale the reference object to 66% of the measurement volume.

**Table 12:** Number of geometry error sources that can be detected at magnification *M*_1_ for configuration 4 for the case of a horizontal plane and a vertical plane when the threshold for sensitivity ratio is 0.594.

Position/Pose Errors (7 Total)			
	Distance Error	Form Error	Distance and Form Error
Horizontal plane	0	1	1
Vertical plane	2	1	3
All planes	2	1	3
Rotation Stage Errors (48 Total)			
	Distance Error	Form Error	Distance and Form Error
Horizontal plane	6	43	43
Vertical plane	6	39	39
All planes	9	43	43

**Table 13:** Number of geometry error sources that can be detected at magnification *M*_1_ for configuration 4 for the case of a horizontal plane and a diagonal plane when the threshold for sensitivity ratio is 0.594.

Position/Pose Errors (7 Total)			
	Distance Error	Form Error	Distance and Form Error
Horizontal plane	0	1	1
Diagonal plane	2	1	3
All planes	2	1	3
Rotation Stage Errors (48 Total)			
	Distance Error	Form Error	Distance and Form Error
Horizontal plane	6	43	43
Diagonal plane	6	40	40
All planes	9	43	43

**Table 14:** Number of geometry error sources that can be detected at magnification *M*_1_ for configuration 4 for the case of a vertical and a diagonal plane, when the threshold for sensitivity ratio is 0.594.

Position/Pose Errors (7 Total)			
	Distance Error	Form Error	Distance and Form Error
Vertical plane	2	1	3
Diagonal plane	2	1	3
All planes	2	1	3
Rotation Stage Errors (48 Total)			
	Distance Error	Form Error	Distance and Form Error
Vertical plane	6	39	39
Diagonal plane	6	40	40
All planes	9	41	41

#### 5.7.5 Observations and Recommendations

**Fig. 8:**
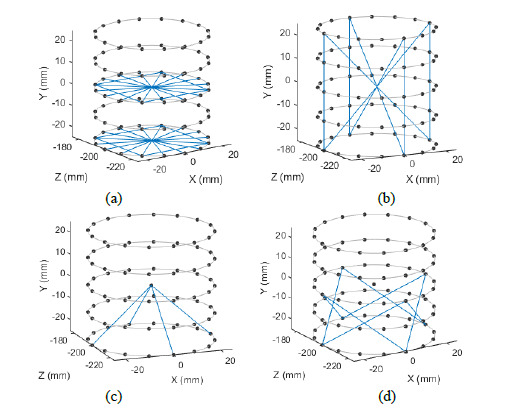
Orientation of test lengths to achieve sensitivity to all detector and rotation stage errors, based on plots shown in Ref. [38]: (a) all face diagonals and lines parallel and perpendicular to the plane of the detector in two horizontal planes, (b) body diagonals and vertical lines, (c) lines joining points in the lowest plane to the center of measurement volume, and (d) lines joining points in the lowest plane to points in the midplane.

Based on the simulations, we make the following observations:

• As noted in Table 4 for magnification *M*_1_, for the nearly ideal case where the reference object consists of many spheres distributed throughout the measurement volume, form error measurements are sensitive to five out of the seven position/pose error sources, so the remaining two error sources—rotation stage *Z* location error and detector rotation about the *X* axis—must be captured through distance error measurements. Rotation stage *Z* location error is important because it affects magnification and therefore will result in scaling errors. Distance error measurements are also sensitive to five out of the seven position/pose errors. The remaining two error sources—detector location error along *X* and detector rotation error about *Z*—must be captured through form error measurements. In the case of rotation stage errors, 32 out of the 48 error sources can be detected through distance error measurements, and all 48 can be detected through form error measurements. This represents the ideal case, *i.e.*, with an almost optimal reference object designed to detect geometry errors at their maximum sensitivity.
• The observation that form error measurements capture more geometry error sources than distance error measurements also holds true for the four testing configurations chosen in this study; see Tables 5–14. However, as mentioned in Sec. 5.5, we note that form error measurements are affected by outliers and therefore are not as reliable as distance error measurements. Also, in practice, it might not be possible to obtain point-cloud data over the entire surface of a sphere because of the way it is mounted on the support structure, which may affect form error measurements.
• The simulations clearly show that scaling the reference object to fill the entire measurement volume achieves the desired testing objective of being sensitive (at a sensitivity ratio of 0.9 or higher) to all 55 geometry error sources. A reference object scaled to 66% and centered in the measurement volume can only detect about 1/10th of the geometry error sources at the maximum sensitivity. This is because, for several error sources, the length/form errors due to the geometry errors do not scale proportionately with the size of the reference object when the object is centered in the volume.
• In the case of form error testing, many geometry error sources require the placement of the sphere as far away from the axis of rotation as possible and in the top or bottom plane. Scaling the reference object to 66% of the volume pushes the sphere closer to the axis of rotation and away from the top or bottom plane, thus reducing the sensitivity to those error sources.
• We showed in Table 4 for magnification *M*_1_ that 32 out of the 48 rotation stage error sources may be detected through distance error measurements, but as shown in Table 5, only 24 out the 48 are detected in configuration 1. Adding measurement lines that are rotationally oriented about the axis of the reference object will provide sensitivity to additional error sources. In Ref. [38], we presented the orientations of lines that provide sensitivity to various geometry error sources. This is also shown in Fig. 8, where the X-ray source is located at the origin (0,0,0), and the detector is located at *Z* = −600 mm. This figure shows face diagonals, body diagonals, and vertical lines oriented at different angles about the central axis in Fig. 8(a) and (b). Other lines include those that join points in the lowest plane to the midplane in Fig. 8(c) and (d). The arrangement of lines in the three planes shown in Fig. 5 only provides some of the lines shown in Fig. 8, thus limiting the sensitivity to some error sources.

We make the following recommendations for testing purposes:

• Full volume reference objects are clearly desirable; *i.e.*, configurations 1 and 3 are optimal for testing purposes.
• In the case of testing configuration 1 (full volume, three planes at magnification *M*_1_ and one plane at magnification *M*_2_), a vertical plane is a better choice for the second magnification when compared to a horizontal or diagonal plane.
• In the case of testing configuration 3 (full volume, two planes at magnification *M*_1_ and two planes at magnification *M*_2_), a horizontal plane and a vertical plane or a horizontal plane and a diagonal plane are desirable at magnification *M*_1_; *i.e.*, a vertical plane and diagonal plane choice is not desirable. At magnification *M*_2_, it is preferable to have planes in two different orientations; *i.e.*, two diagonal planes are not desirable.
• Testing configurations 2 and 4 (reference object occupying 66% of the measurement volume) provided poor sensitivity to most error sources.

## 6 Conclusions

The ASME B89.4.23 standard for performance evaluation of XCT systems for dimensional measurements was released in early 2021. While the standard provides some core testing requirements, there is also some flexibility, allowing for a number of testing configurations that meet the requirements of the standard. In this context, we evaluated four testing configurations to determine whether they are sensitive to the different geometry error sources in XCT systems. Choosing a testing configuration that is maximally sensitive to all systematic sources of error is important in ensuring the system meets specifications for measurements made during regular use. Based on the simulation study, we recommend two testing configurations where the reference object occupies the full measurement volume. The first recommended configuration involves three mandatory planes (horizontal, vertical, and diagonal) at magnification *M*_1_ and a vertical plane at magnification *M*_1_. The second recommended configuration involves a horizontal plane and a vertical plane or a horizontal plane and a diagonal plane at magnification *M*_1_ and any two differently oriented planes at magnification *M*_2_ (for example, a vertical plane and a diagonal plane but not two diagonal planes). Choosing a reference object that only occupies 66% of the measurement volume provides poor sensitivity to the different geometry errors and is not recommended.
